# Supplementary material for: Design and Synthesis of a New Soluble Natural β-Carboline Derivative for Preclinical Study by Intravenous Injection
Source: Int J Mol Sci. 2019 Mar 25;20(6):1491. doi: 10.3390/ijms20061491 (PMC6471559; doi:10.3390/ijms20061491)
Supplement: Supplementary file 1 [file ijms-20-01491-s001.pdf]

Supplementary

CCDC deposition numbers: 1896801-1896804

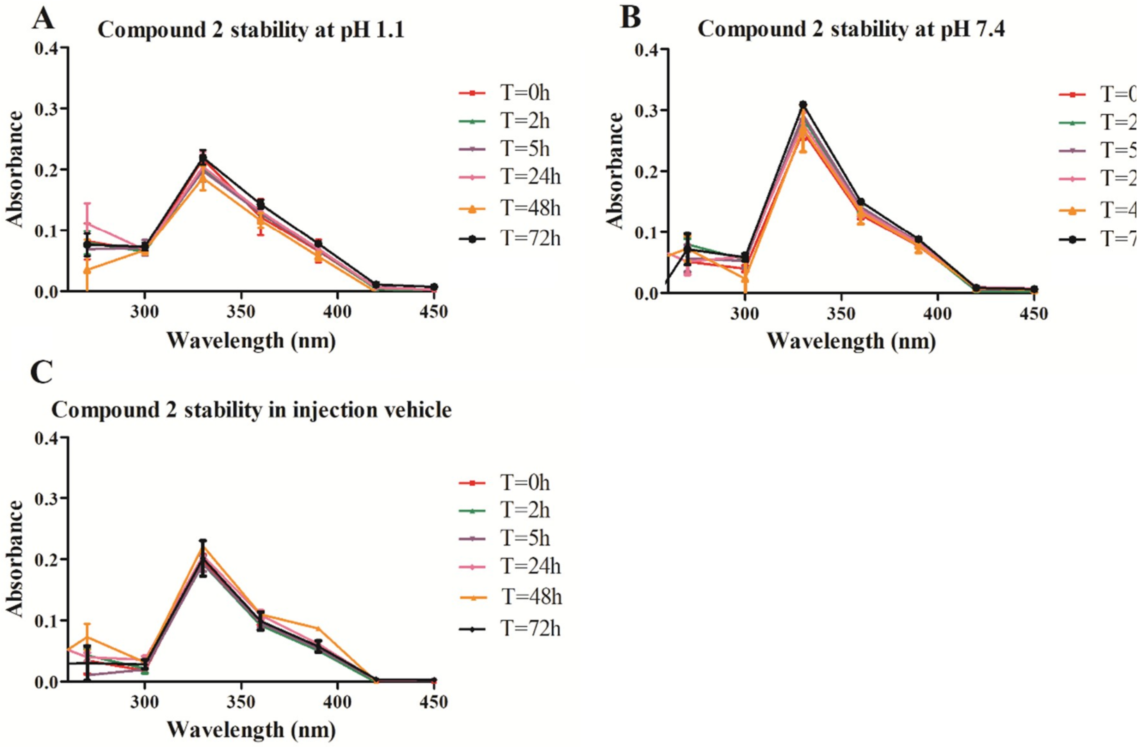

Figure S1. A to C: Compound **2** stability for 72 h at 37°C in pH 1.1, in pH 7.4 and in an injection vehicle composed of 0.9% NaCl and 0.1% Polysorbate 80 pH 7.4.

Table S1. Mechanosynthesis conditions for compounds **2** synthesis with associated yield after purification with Flash Chromatography. BrMePyr = Bromomethylpyridine.

Mechanosynthesis conditions	Yield (%)
Classical synthesis or microwave synthesis	0% product
Neat grinding, no base, 1 eq. 2-BrMePyr	3%, pure compound
1 eq. Na_2CO_3 + 1 eq. 2-BrMePyr, LAG EtOH	20.8%, pure compound
1 eq. Na_2CO_3 + 1 eq. 2-BrMePyr, LAG MeOH	48.4%, pure compound

Table S2. SCXRD, experimental details for harmol and compounds **1a**, **1b** and **2**.

	Harmol	**1a**	**1b**	**2**
**Crystal data**				
Chemical formula	$C_{12}H_{11}N_2O^{+}\cdot Br^{-}\cdot H_2O$	$C_{17}H_{20}N_2O$	$C_{20}H_{26}N_2O$	$C_{26}H_{32}N_3O^{+}Br^{-}$ 0.127(O)
M_r	297.15	268.35	310.43	484.48
Crystal system, space group	Orthorhombic, *Pnma*	Trigonal, $R\bar{3}:H$	Monoclinic, $P2_1/c$	Triclinic, $P\bar{1}$
a, *b*, *c* (Å)	13.3202(3), 6.6792(3), 13.8950(4)	23.5683(3), 23.5683(3), 14.8335(2)	9.1898(2), 10.3178(3), 18.6658(4)	9.3131(3), 12.0050(6), 12.5274(6)
α, β, γ (°)	90, 90, 90	90, 90, 120	90, 92.261(2), 90	72.123(4), 85.718(3), 74.778(3)
V (Å^3)	1236.21(7)	7135.6(2)	1768.49(7)	1286.19(10)
Z	4	18	4	2
Radiation type	Mo $K\alpha$	Cu $K\alpha$	Mo $K\alpha$	Mo $K\alpha$
μ (mm^{-1})	3.32	0.55	0.07	1.62
Crystal size (mm)	0.40 × 0.17 × 0.13	0.55 × 0.20 × 0.15	0.45 × 0.37 × 0.28	0.46 × 0.34 × 0.09
**Data collection**				
Absorption correction	Analytical[1]	Analytical[1]	Analytical[1]	Analytical[1]
T_{min}, T_{max}	0.467, 0.780	0.835, 0.932	0.975, 0.984	0.599, 0.888
No. of measured, independent and observed [$I > 2\sigma(I)$] reflections	4996, 1654, 1123	8192, 2786, 2581	9756, 3903, 3134	13828, 6928, 5530
R_{int}	0.021	0.015	0.016	0.023
$(\sin \theta/\lambda)_{max}$ (Å^{-1})	0.667	0.597	0.641	0.685
**Refinement**				
$R[F^2 > 2\sigma(F^2)]$, $wR(F^2)$, S	0.027, 0.067, 0.91	0.043, 0.128, 1.06	0.043, 0.124, 1.03	0.038, 0.110, 1.03

No. of reflections	1654	2786	3903	6928
No. of parameters	108	188	289	295
No. of restraints	2	0	244	6
H-atom treatment	H atoms treated by a mixture of independent and constrained refinement	H atoms treated by a mixture of independent and constrained refinement	H-atom parameters constrained	H-atom parameters constrained
$\Delta\rho_{max}$, $\Delta\rho_{min}$ (e Å^{-3})	0.39, -0.46	0.20, -0.16	0.17, -0.19	0.31, -0.25

[1]*CrysAlis PRO*, Oxford Diffraction Ltd., Versions 1.171.33.55 (Oxford Diffraction Ltd., 2010) and 1.171.39.46 (Rigaku OD, 2018) Analytical numeric absorption correction using a multifaceted crystal model based on expressions derived by R.C. Clark & J.S. Reid. (Clark, R. C. & Reid, J. S. (1995). Acta Cryst. A51, 887-897)

Computer programs: *CrysAlis PRO* 1.171.33.55 (Oxford Diffraction Ltd., 2010), *CrysAlis PRO* 1.171.39.46 (Rigaku OD, 2018), *SHELXT* (Sheldrick, 2015) [1] ,*SHELXL 2018*/3 (Sheldrick, 2016) [2-4], *SHELXL2016*/6 (Sheldrick, 2016) [2-4].

References

1. Sheldrick, G.M. SHELXT - Integrated space-group and crystal-structure determination. *Acta Crystallogr. Sect. A Found. Crystallogr.* **2015**, *71*, 3–8.
2. Sheldrick, G.M. Crystal structure refinement with SHELXL. *Acta Crystallogr. Sect. C Struct. Chem.* **2015**, *71*, 3–8.
3. Dolomanov, O. V.; Bourhis, L.J.; Gildea, R.J.; Howard, J.A.K.; Puschmann, H. OLEX2: A complete structure solution, refinement and analysis program. *J. Appl. Crystallogr.* **2009**, *42*, 339–341.
4. Hübschle, C.B.; Sheldrick, G.M.; Dittrich, B. ShelXle : a Qt graphical user interface for SHELXL. *J. Appl. Crystallogr.* **2011**, *44*, 1281–1284.
